# Inflammatory Biomarkers in Exhaled Breath Condensate: A Systematic Review

**DOI:** 10.3390/ijms23179820

**Published:** 2022-08-29

**Authors:** Federica Ghelli, Marco Panizzolo, Giacomo Garzaro, Giulia Squillacioti, Valeria Bellisario, Nicoletta Colombi, Enrico Bergamaschi, Irina Guseva Canu, Roberto Bono

**Affiliations:** 1 Department of Public Health and Pediatrics, University of Turin, 10126 Turin, Italy; 2 Federated Library of Medicine “F. Rossi”, University of Turin, 10126 Turin, Italy; 3 Center for Primary Care and Public Health (Unisanté), University of Lausanne, 1066 Lausanne, Switzerland

**Keywords:** inflammation, cytokines, exhaled breath condensate, non-invasive, reference values, non-smoking healthy adults

## Abstract

Inflammation is a comprehensive set of physiological processes that an organism undertakes in response to a wide variety of foreign stimuli, such as viruses, bacteria, and inorganic particles. A key role is played by cytokines, protein-based chemical mediators produced by a broad range of cells, including the immune cells recruited in the inflammation site. The aim of this systematic review is to compare baseline values of pro/anti-inflammatory biomarkers measured in Exhaled Breath Condensate (EBC) in healthy, non-smoking adults to provide a summary of the concentrations reported in the literature. We focused on: interleukin (IL)-1β, IL-4, IL-6, IL-8, IL-10, tumour necrosis factor-alpha (TNF-α), and C reactive protein (CRP). Eligible articles were identified in PubMed, Embase, and Cochrane CENTRAL. Due to the wide differences in methodologies employed in the included articles concerning EBC sampling, storage, and analyses, research protocols were assessed specifically to test their adherence to the ATS/ERS Task Force guidelines on EBC. The development of reference intervals for these biomarkers can result in their introduction and use in both research and clinical settings, not only for monitoring purposes but also, in the perspective of future longitudinal studies, as predictive parameters for the onset and development of chronic diseases with inflammatory aetiology.

## 1. Introduction

Inflammation is a comprehensive set of physiological processes that an organism undertakes in response to a foreign stimulus, including human pathogens, such as viruses and bacteria, and inorganic particles [1]. Depending on the duration of these processes, it is possible to distinguish between two inflammatory response types: acute and chronic [2]. In both cases, a key role is played by cytokines, protein-based chemical mediators produced by a broad range of cells, including the immune cells recruited in the inflammation site. These polypeptides are pleiotropic molecules that elicit their effects in an autocrine or paracrine manner, binding to specific receptors on cell walls and regulating their activation [3]. Cytokines can be classified according to their role as pro-inflammatory, anti-inflammatory, or chemotactic. The pro-inflammatory cytokines owe their name to their role in orchestrating the early immune response to infection/injury by recruiting immune cells to the infection site and activating them [4]. They are often released in a cascade, and the lack of control over their release/activity can lead to damage to host tissues as well as pathogens [4]. The main cytokines with a pro-inflammatory role are interleukin (IL)-1β, IL-6, and tumour necrosis factor α (TNF-α). Anti-inflammatory cytokines, instead, such as IL-4 and IL-10, play a crucial role in controlling the regulation of pro-inflammatory cytokines. Finally, chemokines are a cytokine subgroup whose main role is the activation and recruitment of leukocytes, as, for instance, monocyte chemoattractant protein-1 (MCP-1), macrophage inflammatory protein (MIP)-1a, MIP-1b and IL-8 [5]. Another non-cytokine polypeptide, named C-reactive protein (CRP), is an acute inflammatory protein that increases its concentration at sites of inflammation or infection [6]. It may be considered a useful diagnostic tool in the assessment of early inflammation, such as in acute-phase diseases [7]. Most biomarkers of inflammation and oxidative stress (OS) are often investigated in clinical settings using invasive biological matrices, such as blood and broncho-alveolar lavage (BAL).

Molecular epidemiology studies, especially when involving children and the elderly, can reliably rely on biological matrices collected by non-invasive methods such as Exhaled Breath Condensate (EBC) and urine [8,9]. Cytokine profiling analyses play a crucial role in the early detection and follow-up of inflammatory processes. Among non-invasive matrices, EBC is a validated method for assessing volatile markers and inflammatory mediators. This methodology allows collecting droplets from airway lining fluid by the condensation of warm, humid breath onto a cold surface in a condensing device [10]. To date, a variety of both commercial and homemade devices for the collection of EBC are available. The most widely used commercial devices are EcoScreen™, RTube™, and TurboDECCS™ [8]. The samplers differ in the cooling system type (pre-cooled sleeve or electric cooling system), providing temperatures ranging from 0 °C to −20 °C in the tube covering materials and in the electrical power [11]. In non-clinical studies, there is a greater effort to provide standardisation of non-invasive sampling methods and to provide reference values of OS and inflammation biomarkers in the general population, with the purpose of identifying a range that can highlight a possible onset of disease [12]. Therefore, the aim of this systematic review is to compare baseline values of pro/anti-inflammatory biomarkers measured in EBC in healthy, non-smoking adults to provide a summary of the concentrations reported in the literature. A further goal is to highlight possible methodological issues preventing the definition of reference intervals, to employ them not only in clinical scenarios but even in environmental and occupational settings. We focused on the most searched biomarkers quantified in EBC: interleukin 1β (IL-1β), interleukin 4 (IL-4), interleukin 6 (IL-6), interleukin 8 (IL-8), interleukin IL 10 (IL-10), tumor necrosis factor-alpha (TNF-α) and C reactive protein (CRP).

## 2. Materials and Methods

The present systematic review protocol is registered on PROSPERO database (Protocol ID = CRD42022316248). The registration underwent only the basic automated checks for eligibility to enable the PROSPERO team to focus on COVID-19 submissions. The study is reported in accordance with the PRISMA 2020 Statement [13].

### 2.1. Study Selection

Eligible articles were searched and identified in PubMed, Embase, and Cochrane CENTRAL up to 4 February 2022.

The search string aimed to find original research articles evaluating the concentration of some inflammatory biomarkers in EBC, including the following terms: “Cytokines”, “Interleukins”, “C-Reactive Protein”, “Interleukin-1”, “Interleukin-4”, “Interleukin-6”, “Interleukin-8”, “Interleukin-10”, “Tumor Necrosis Factor-alpha”, “exhaled breath condensate*”. Full strings are reported in Appendix A (Table A1). Table 1 summarises the pathophysiological role of these biomarkers.

### 2.2. Inclusion and Exclusion Criteria

Observational or interventional original research studies on healthy humans (18+ years, non-smoking, no known disease) measuring the selected biomarkers in EBC were considered potentially eligible. Only full texts written in English were considered suitable for inclusion.

Non-quantitative data, full texts with unpublished data, reviews, non-human and in vitro studies, correspondence, conferences, abstracts without full text, expert opinions, protocols, and editorials were excluded.

**Figure 1:**
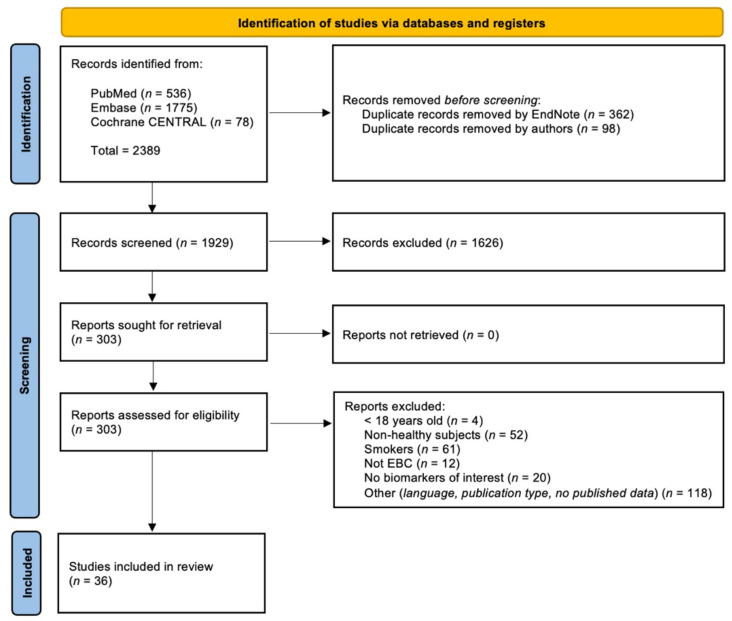
PRISMA flow chart summarising the study selection process.

Two reviewers completed the article selection in blind process, screening titles and abstracts according to the inclusion and exclusion criteria declared. In case of insufficient data, the selection was based on the full texts. Disagreements on article selection were discussed and eventually submitted to a third reviewer. The procedure is summarised in the PRISMA diagram [13] reported in Figure 1.

### 2.3. Data Extraction

Two researchers independently extracted the data from the selected articles by filling in a spreadsheet. We reported the following information: author’s name, publication time, title, country, study design, recruitment method, number of subjects, subject category, inclusion and exclusion criteria, male (n°), female (n°), age, BMI, timing (pre- and post-intervention), collection details (device, temperature, and time), storage temperature, α-amylase control, analytical methods, biomarker concentrations, Limit of Detection (LOD), main results and notes. Data reported by graphs in original studies were extracted by the WebPlotDigitizer software (Rohatgi (2021), version 4.5, Pacifica, California, USA, https://automeris.io/WebPlotDigitizer/ accessed on 25 July 2022).

### 2.4. Quality Assessment

The quality assessment of the included articles was performed by two independent reviewers in a two-step process. The first part of the assessment was carried out according to the study design by the proper Joanna Briggs Institute (JBI) checklists to assess the reliability and relevance of the published articles. The second part was focused on the methodological protocol, specifically to test the adherence of the research protocols to the ATS/ERS Task Force guidelines on Exhaled Breath Condensate [20,21]. The checklist is reported in Appendix B (Table A2). Each study was awarded a Completeness of Reporting (COR) score according to the number of items met in each of the two checklists employed. The score was calculated as COR (%) = (“satisfied”/(“satisfied” + “not satisfied/unclear”)) × 100). Quality was then defined as “poor” (COR < 50%), “moderate” (COR = 50–74%) or “high” (COR ≥ 75%) [22]. The final ranking due to each checklist has been kept separate for each of the included studies. Any discrepancy between reviewers was discussed, and if required, a third reviewer was consulted.

### 2.5. Statistical Analysis

Categorical variables have been reported as frequency (*n*), while continuous variables were reported as Mean ± Standard Deviation (SD) or Mean ± Standard Error of the Mean (SEM) or Median and Interquartile Range (IQR), as reported in the original research articles. For studies declaring the analytical LOD, arithmetic mean and SE of data above this parameter were approximated in order to obtain a graphical representation [23]. The forest plot was created by R Studio (RStudio Team (2020). RStudio: Integrated Development for R. RStudio, PBC, Boston, MA, USA).

## 3. Results

### 3.1. Qualitative Synthesis

Among the 2389 items initially identified, 460 duplicates were removed before screening by EndNote and manually. The remaining 1929 were screened, and 36 research articles were included in the systematic review [7,24,25,26,27,28,29,30,31,32,33,34,35,36,37,38,39,40,41,42,43,44,45,46,47,48,49,50,51,52,53,54,55,56,57,58]. The exclusion criteria lead to the removal of 267 articles. Among these, 117 papers were excluded because of the epidemiological sample characteristics (juveniles subjects (*n* = 4), non-healthy subjects (*n* = 52), smoking subjects (*n* = 61)), 12 because they did not include the EBC matrix, 20 for not assessing the biomarkers included in the string, and 118 were excluded because they were not in English, they were not research articles, or they had a lack of data. The procedure is summarised in the PRISMA diagram reported in Figure 1.

### 3.2. Study and Participant Characteristics

**Figure A1:**
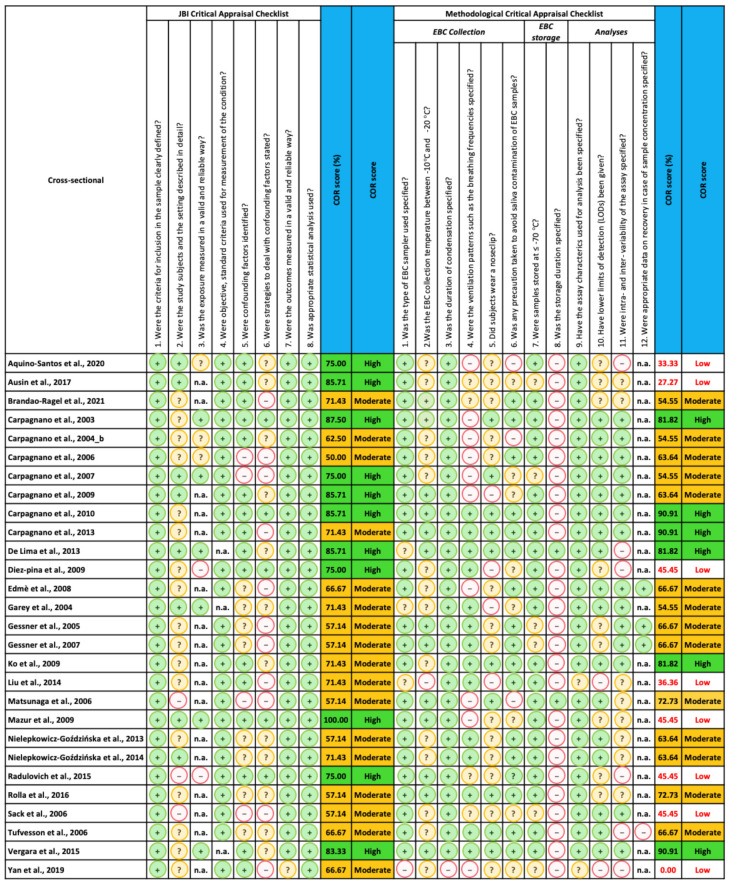
Quality Assessment for cross-sectional studies, according to JBI critical appraisal tool and to the ATS/ERS Task Force guidelines on EBC [20,21]. (+) means “yes”; (-) means “no”; (?) means “unclear”; (n.a.) means “not applicable”.

**Figure A2:**
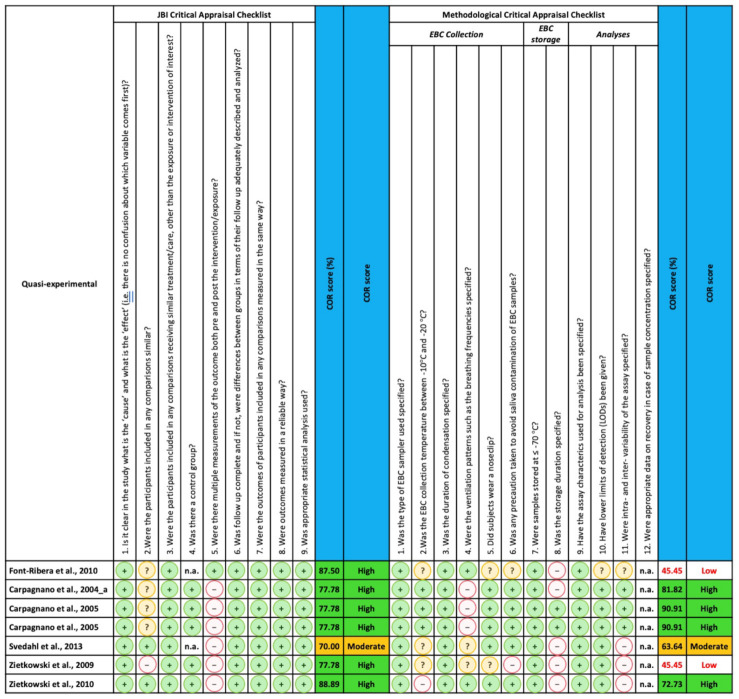
Quality Assessment for quasi-experimental studies, according to JBI critical appraisal tool and to the ATS/ERS Task Force guidelines on EBC [20,21]. (+) means “yes”; (-) means “no”; (?) means “unclear”; (n.a.) means “not applicable”.

**Figure A3:**
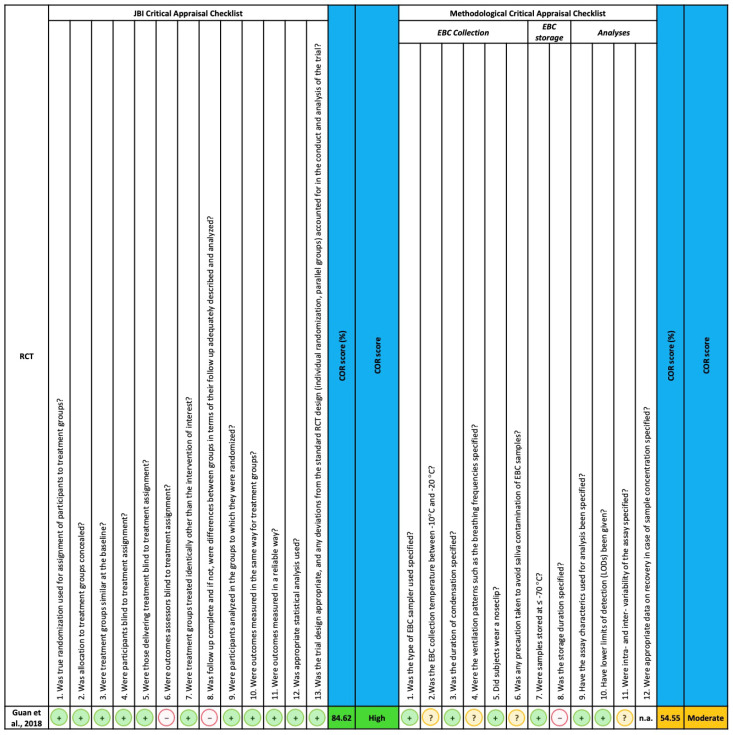
Quality Assessment for randomised-control trial studies, according to JBI critical appraisal tool and to the ATS/ERS Task Force guidelines on EBC [20,21]. (+) means “yes”; (-) means “no”; (?) means “unclear”; (n.a.) means “not applicable”.

Appendix C reports the quality assessment scores (Figure A1, Figure A2 and Figure A3). All the included studies were assessed by adopting the proper JBI checklists according to the study design (cross-sectional studies (28), quasi-experimental studies (7), and randomised controlled trials (1)). A total of 50% of the studies were awarded a “High” quality score, while 50% with a “Moderate” quality score. Furthermore, due to the lack of questions assessing the methodological approach in those tools, we created an additional checklist for the objective assessment of the analytical methods applied in the included studies. According to this second evaluation, 10 of the studies were awarded a “High” quality score, 16 with a “Moderate” quality score, and 10 with a “Low” quality score.

### 3.3. Inflammation Biomarkers in EBC

Table 2 reports the characteristics of the studies specifying the absence or presence of LOD and, in this case, the percentage of determinations above the assay sensitivity.

**Figure 2:**
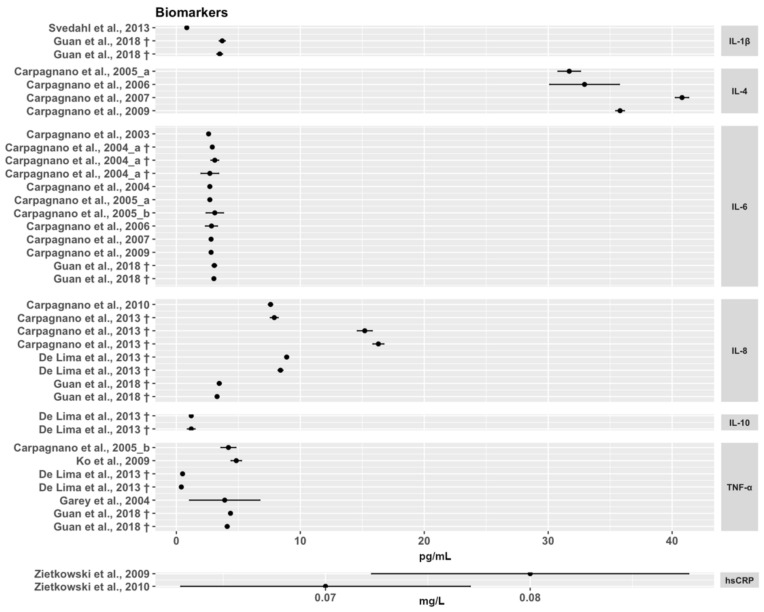
Forest plot summarising the concentration of the selected biomarkers in the articles where the sensitivity of the employed assays, and the measurements above the LOD were reported. † More subjects groups were analysed in the same article. The “a” and “b” following the indication of articles with the same first author and year are referred to the order of the articles in the bibliography paragraph [7,27,28,29,30,31,32,33,34,35,36,37,41,44,45,54,58].

The forest plot (Figure 2) summarises the biomarker concentrations reported in papers declaring the assay LOD and the measurements above it. The values measured in Edmè et al., 2008 have not been included because the concentration declared was not divided by the concentration factor. As well, we did not include the quantification assessed by Matsunaga et al., 2006 because the authors reported only the relative intensity concentrations expressed as percentages. The concentrations extracted are reported in Table 3, while the details of data reported in those articles not declaring the assay LOD or reporting measurements lower than this parameter are reported in Supplementary Materials (Table S1).

## 4. Discussion

The analysis of inflammatory biomarkers in EBC in both occupational and environmental studies is increasingly topical. The primary aim of the selected papers was to detect early changes in airway inflammatory status that could be related to a higher risk of developing pulmonary disorders [30]. The lack of established reference values in the general healthy non-smoking population, however, makes such achievement difficult.

Despite the easiness and non-invasiveness of sampling, our review highlights the lack of a standardised analytical protocol among researchers, making any inter-studies comparison challenging. These issues mainly concern the criteria used when selecting groups in epidemiological studies, sampling and storage protocols, as well as the comparability of analytical methods and eventual pre-treatment procedures.

Therefore, we established to carry on the quality assessment not only on the basis of the study design but also on a detailed evaluation of their methodological quality. The most common critical issue highlighted by the JBI checklists concerns the lack of a detailed description of subjects enrolled, with the subsequent poor characterisation of eventual confounding factors able to influence their inflammatory status. Obesity, for example, is associated with both systemic and airway inflammation [27]. Even though the underlying mechanisms have not been clearly elucidated and contrasting results have been reported, some authors suggest that the release of cytokines by the adipose tissue may be related to respiratory disorders such as obstructive sleep apnea syndrome (OSAS), obesity hypoventilation syndrome (OHS), asthma or chronic obstructive pulmonary disease (COPD) [59,60,61,62]. Only 16 of the included studies reported the BMI of the subject enrolled. Indeed, most of the studies included in the present review consist of small age-matched control groups from clinical studies, who are described only as healthy and non-smokers. Airways or systemic inflammation can increase with ageing [63]; thus, a detailed characterisation of this status should be performed in subgroups of the population using EBC, which allows repeated measurements over time [28].

The methodological assessment was based on compliance with the guidelines issued by the American Thoracic Society/European Respiratory Society Task Force in 2005 and 2015 [20,21]. To date, some of the critical issues highlighted are still unsolved. Concerning the EBC collection, the characteristics of the collection device may influence the biomarker concentration in the final sample [64]. In our systematic review, most of the articles included using Ecoscreen™ sampling devices. In many studies, the ventilation pattern sustained by subjects during the sampling is not declared, despite the importance of sampling during tidal breathing to avoid an alteration in the biomarker composition, especially for those biomarkers that may be sensitive to the respiratory pattern [65]. Inflammatory markers are produced in both the airway and the alveolar compartments, defining, at least partially, a possible flow-rate dependence of their concentration in EBC [66].

Wearing a nose clip was often not reported or not in use (56%). Albeit slightly uncomfortable, it is recommended to minimise the contamination with the nasal airway lining fluid and make subjects exhale strictly through the mouth [20]. The salivary contamination, which could determine a contribution to the inflammatory biomarker levels in EBC, was generally prevented by saliva-trap on sampling devices or by mouth rinses before the sampling. Some researchers also quantified the amylase levels, even though this method can be affected by some false positives [20]. Concerning the EBC storage, on the contrary, the vast majority of the included studies did not report the duration of the sample storage, assuming the concentration of cytokines remained stable over time. In frozen plasma samples, most cytokines are stable for up to two years, with the exception of IL-1β, IL-6, and IL-10, which undergo a degradation process up to 50% within 2–3 years of storage [67]. Further studies aiming to assess the cytokine stability in EBC would thus be recommended.

The main critical issue in the quantification of inflammatory biomarkers levels, however, concerns the analytical methods. Cytokines in EBC are often quantified by ELISA or Cytometric Bead Array (CBA) assays, according to the manufacturer’s guidelines. However, as previously pointed out by Horvath et al., EBC is a diluted matrix and the cytokine concentration is generally around the assay LOD, where assay variability is higher. Information about the assay validation for this matrix or any reason justifying the assay choice was generally not provided. Moreover, 33% of the articles did not report the assay LOD declared by the manufacturers, whereas in some cases, the quantification declared was lower than the assay LOD. The lack of this information significantly affects the reliability of these measurements, preventing the possibility of comparing data with those obtained from other studies. In both cases, we considered those data as potentially biased, and thus we excluded them from the summarising forest plot. The assays, indeed, appear to be more sensitive in discriminating large differences in cytokine levels due to acute vs chronic inflammatory states, while in healthy conditions, smaller magnitudes of cytokine levels were observed [34]. In some studies, EBC was concentrated lyophilising samples to improve the assay performance, despite this being a complex and expensive method [68]. This methodology could be a source of bias when comparing data from different studies.

Another current critical issue is the normalisation of biomarker levels in EBC to take into account the inter-individual variability in droplet formation, resulting in samples being variously diluted. To overcome this problem, in some studies, data were reported both raw and normalised for the total protein concentration in EBC, even if this is not a widely accepted method [39,42,43]. Moreover, EBC collection involves a large variability in the volume exhaled for each breath over time. Thus, the American Thoracic Society (ATS) has suggested standardising the concentrations of biomarkers in EBC by registering the total volume of exhaled air and stopping the exhalation collection when the set volume has been accomplished. Thus, EBC collection will consider the volume of exhaled breath, the volume of condensation collected from the exhaled volume, and the collection time must be correlated in order to evaluate the effectiveness of the collection of EBC. To achieve this goal, a volume-meter can be enclosed in line with the DECCS circuit, thus allowing measuring the total volume of air exhaled (e.g., 90 L) during an EBC collection session.

To provide a complete description of the more studied inflammatory mediators measured in EBC, we focus on IL-1β, IL-4, IL-6, IL-8, IL-10, TNF-α, and CRP (as determined by the high sensitive assay).

The data retrieved in this review present some limits, actually preventing the possibility of considering them as truly reference values. First of all, the vast majority of the selected studies describe small epidemiological samples representing the control group in clinical studies, an aim that does not match the purpose of our review. The frequent absence of a detailed description of those subjects in terms of demographic and health-related data hampers the analysis of sources of variability in biomarker concentration, which would inform the need for partitioning when summarising the reference values and the reference interval. Secondly, methodological discrepancies and the lack of standardisation in sampling and analysis protocols make it difficult to compare data obtained in different settings.

The strength of our systematic review can thus be identified in the research string that results are very sensitive, even though non-specific, allowing us to obtain a comprehensive set of articles to screen and to highlight the main criticisms still affecting the evaluation of the inflammatory profile in EBC.

## 5. Conclusions

In conclusion, EBC is a useful tool to characterise the airway inflammatory state due to the easiness and non-invasiveness of sampling. However, to obtain consistent reference values, more efforts are needed. Firstly, the creation of datasets with measurements obtained from vast epidemiological samples suitably selected according to health criteria and with repeated measurements would be strongly recommended. Secondly, qualitative criteria requested from the study design must be integrated with the criteria proposed by the ATS/ERS Task Force guidelines on Exhaled Breath Condensate in 2005 and 2015 [20,21,68].

The development of reference intervals for these biomarkers can result in their introduction and use in both research and clinical settings, not only for monitoring purposes but also, in the perspective of future longitudinal studies, as a predictive parameter for the onset and development of chronic diseases with inflammatory aetiology.

## Figures and Tables

**Table 1:** Most searched biomarkers in EBC.

Biomarkers	Role	Description
CRP	Pro-inflammatory	Detection of bacteria and damaged human cells and complement activation. Circulating concentration rises in response to infection and is associated with risk of coronary heart disease [6].
IL-1β	Pro-inflammatory	Response to exogenous and endogenous noxious stimuli and induction of IL-6 and IL-8 secretion by bronchial epithelial cells [14,15].
IL-4	Anti-inflammatory	Response to allergic airway inflammation [16].
IL-6	Pro-inflammatory	Response to several stimuli, including exercise, allergens, and respiratory viruses [5].
IL-8	Pro-inflammatory	Neutrophil recruitment with an important role in pathological and physiological conditions [15,17].
IL-10	Anti-inflammatory	Immune-suppressive cytokine, which reduces the recruitment of effector T cells and counteracts the effects of TNF-α and IL-1β Response to allergic challenge [18].
TNF-α	Pro-inflammatory	Pleiotropic immune activator, involved in many airway disorders [19].

**Table 2:** Frequency of studies reporting or not reporting value above the LOD. Some studies analysed more than one biomarker.

Biomarker	n° of Studies	n° of Studies (%) with Data > LOD	n° of Studies (%) with Data < LOD	n° of Studies (%) without LOD Declared
CRP	3	2 (66.7%)	-	1 (33.3%)
IL-1β	12	2 (16.7%)	5 (41.7%)	5 (41.7%)
IL-4	11	6 (54.5%)	2 (18.2%)	3 (27.3%)
IL-6	19	11 (57.9%)	2 (10.5%)	6 (31.6%)
IL-8	16	5 (31.3%)	4 (25.0%)	7 (43.8%)
IL-10	12	2 (16.7%)	2 (16.7%)	8 (66.7%)
TNF-α	18	6 (33.3%)	3 (16.7%)	9 (50.0%)

**Table 3:** Data extracted from articles reporting data above the declared assay LOD. Data are expressed as: Geometric mean = †; Mean ± SD; Median (IQR); Median (“25°th–75°th”); Median [min–max].

Authors, Year	Country	n° Subjects (M;F)	Age	Collection Device	Collection Temperature	Storage Temperature	Analytical Method	Data	LOD	SCORE Quality Assessment JBI	SCORE Authors’ Quality Assessment
**CRP**											
Zietkowski et al., 2009 [7]	Poland	15 (6;9)	33.13 (6.71) †	EcoScreen; Eric Jaeger GmbH, Hoechberg, Germany	0 °C	−80 °C	highly sensitive CRP assay (Konelab, Waltham, MA, USA)	0.08 ± 0.03 mg/L	0.05 mg/L	77.78 High	45.45 Low
Zietkowski et al., 2010 [58]	Poland	8 (4;4)	29.9 (7.1) †	EcoScreen; Eric Jaeger GmbH, Hoechberg, Germany	0 °C	−80 °C	highly sensitive CRP assay (Konelab, Waltham, MA, USA)	0.07 ± 0.03 mg/L	0.02 mg/L	88.89 High	72.73 High
**IL-1β**											
Guan et al., 2018 [44]	China	15 (7;8)	20 ± 1	ECOScreen (Jager, Germany)	NA	−80 °C	BD Cytometric Bead Array, BD-Biosciences, San Jose, CA, USA	3.71 (2.31) pg/mL	2.4 pg/mL	84.62 High	54.55 Medium
Guan et al., 2018 [44]	China	15 (7;8)	20 ± 1	ECOScreen (Jager, Germany)	NA	−80 °C	BD Cytometric Bead Array, BD-Biosciences, San Jose, CA, USA	3.34 (2.26) pg/mL	2.4 pg/mL	84.62 High	54.55 Medium
Svedahl et al., 2013 [54]	Norway	24 (14;10)	23.8 ± 2.5	ECoScreen; Jager, Wurzburg, Germany	NA	−70 °C	Quantikine HS from R&D Systems (Minneapolis, MN, USA)	0.84; CI= 0.64–1.10 pg/mL	0.05 pg/mL	77.78 High	63.64 Medium
**IL-4**											
Carpagnano et al., 2005_a [30]	Italy	15 (5;10)	35 ± 6	EcoScreen (Jaeger, Wurzburg, Germany)	−20 °C	−70 °C	EIA (Cayman Chemical, Ann Arbor, MI, USA)	31.7 ± 3.5 pg/mL	20 pg/mL	77.78 High	90.91 High
Carpagnano et al., 2006 [32]	Italy	17 (8;9)	37 ± 9	EcoScreen (Jaeger, Wurzburg, Germany)	On ice	−70 °C	EIA (Cayman Chemical, Ann Arbor, MI, USA)	31.6 (27.5–39.7)pg/mL	20 pg/mL	50.00 Medium	63.64 Medium
Carpagnano et al., 2007 [33]	Italy	10 (5;5)	44 ± 8	EcoScreen (Jaeger, Wurzburg, Germany)	NA	−70 °C	EIA (Cayman Chemical, Ann Arbor, MI, USA)	40.8 ± 1.7 pg/mL	15 pg/mL	75.00 High	54.55 Medium
Carpagnano et al., 2009 [34]	Italy	10 (-;-)	43 ± 9	EcoScreen (Jaeger, Wurzburg, Germany)	−20 °C	−80 °C	EIA (Cayman Chemical, Ann Arbor, MI, USA)	35.8 ± 1.1 pg/mL	20 pg/mL	85.71 High	63.64 Medium
Edmè et al., 2008 * [39]	France	19 (-;-)	38.3 ± 13.6	EcoScreen (Jaeger, Wurzburg, Germany)	NA	−80 °C	Cytometric Bead Arrays (CBA) Becton Dickinson, San Jose, CA	32.1 (23 76) † pg/mL	5 pg/mL	66.67 Medium	66.67 Medium
Matsunaga et al., 2006 [47]	Japan	10 (3;7)	34.4 ± 6.6	EcoScreen, (Jaeger, Germany)	−20 °C	−70 °C	Human Inflammation Antibody III (ray Biontec Inc, Norcross, GA, USA)	5.2 ± 1.7 pg/mL	1pg/mL	57.14 Medium	72.73 Medium
**IL-6**											
Carpagnano et al., 2003 [27]	Italy	14 (8;6)	45 ± 6	EcoScreen (Jaeger, Wurzburg, Germany)	−20 °C	−70 °C	EIA (Cayman Chemical, Ann Arbor, MI, USA)	2.6 ± 0.2 pg/mL	1.5 pg/mL	87.50 High	81.82 High
Carpagnano et al., 2004_a [28]	Italy	18(5;13)	46 ± 6	EcoScreen (Jaeger, Wurzburg, Germany)	On ice	−70 °C	EIA (Cayman Chemical, Ann Arbor, MI, USA)	2.9 ± 0.6 pg/mL	1.5 pg/mL	77.78 High	81.82 High
Carpagnano et al., 2004_a [28]	Italy	5 (2;3)	47 ± 3	EcoScreen (Jaeger, Wurzburg, Germany)	On ice	−70 °C	EIA (Cayman Chemical, Ann Arbor, MI, USA)	3.1 ± 0.6 pg/mL	1.5 pg/mL	77.78 High	81.82 High
Carpagnano et al., 2004_b [29]	Italy	15 (8;7)	48 ± 7	EcoScreen (Jaeger, Wurzburg, Germany)	−20 °C	−70 °C	EIA (Cayman Chemical, Ann Arbor, MI, USA)	2.7 ± 0.6 pg/mL	1.5 pg/mL	62.50 Medium	54.55 Medium
Carpagnano et al., 2005_a [30]	Italy	15 (5;10)	35 ± 6	EcoScreen (Jaeger, Wurzburg, Germany)	−20 °C	−70 °C	EIA (Cayman Chemical, Ann Arbor, MI, USA)	2.7 ± 0.6 pg/mL	1.5 pg/mL	77.78 High	90.91 High
Carpagnano et al., 2005_b [31]	Italy	7 (5;2)	42 ± 5	EcoScreen (Jaeger, Wurzburg, Germany)	−20 °C	−70 °C	EIA (Cayman Chemical, Ann Arbor, MI, USA)	3.1 ± 0.7 pg/mL	1.5 pg/mL	77.78 High	90.91 High
Carpagnano et al., 2006 [32]	Italy	17 (8;9)	37 ± 9	EcoScreen (Jaeger, Wurzburg, Germany)	On ice	−70 °C	EIA (Cayman Chemical, Ann Arbor, MI, USA)	2.6 (1.9-4.0) pg/mL	1.5 pg/mL	50.00 Medium	63.64 Medium
Carpagnano et al., 2007 [33]	Italy	10 (5;5)	44 ± 8	EcoScreen (Jaeger, Wurzburg, Germany)	NA	−70 °C	EIA (Cayman Chemical, Ann Arbor, MI, USA)	2.8 ± 0.1 pg/mL	1.5 pg/mL	75.00 High	54.55 Medium
Carpagnano et al., 2009 [34]	Italy	10 (-;-)	43 ± 9	EcoScreen (Jaeger, Wurzburg, Germany)	−20 °C	−80 °C	EIA (Cayman Chemical, Ann Arbor, MI, USA)	2.8 ± 0.1 pg/mL	1.5 pg/mL	85.71 High	63.64 Medium
Edmè et al., 2008 * [39]	France	19 (-;-)	38.3 ± 13.6	EcoScreen (Jaeger, Wurzburg, Germany)	NA	−80 °C	Cytometric Bead Arrays (CBA) Becton pg/mL Dickinson, San Jose, CA, USA	111.7 (70-362) † pg/mL	5 pg/mL	66.67 Medium	66.67 Medium
Guan et al., 2018 [44]	China	15 (7;8)	20 ± 1	ECOScreen (Jager, Germany)	NA	−80 °C	BD Cytometric Bead Array, BD-Biosciences, San Jose, CA, USA	3.09 (3.08) pg/mL	2.4 pg/mL	84.62 High	54.55 Medium
Guan et al., 2018 [44]	China	15 (7;8)	20 ± 1	ECOScreen (Jager, Germany)	NA	−80 °C	BD Cytometric Bead Array, BD-Biosciences, San Jose, CA, USA	3.08 (2.03) pg/mL	2.4 pg/mL	84.62 High	54.55 Medium
Matsunaga et al., 2006 [47]	Japan	10 (3;7)	34.4 ± 6.6	EcoScreen, (Jaeger, Germany)	−20 °C	−70 °C	Human Inflammation Antibody III (ray Biontec Inc, Norcross, GA, USA)	5.2 ± 1.2 pg/mL	1 pg/mL	57.14 Medium	72.73 Medium
**IL-8**											
Carpagnano et al., 2010 [35]	Italy	8 (5;3)	42 ± 4	EcoScreen (Jaeger, Wurzburg, Germany)	−20 °C	−70 °C	EIA kit (Human Interleukin-8, Bender med-Systems, Vienna, Austria)	7.6 ± 0.5 pg/mL	1.3 pg/mL	85.71 High	90.91 High
Carpagnano et al., 2013 [36]	Italy	10 (5;5)	26 ± 4.9	EcoScreen (Jaeger, Wurzburg, Germany)	−20 °C	−70 °C	EIA (Cayman Chemical, Ann Arbor, MI, USA)	7.9 ± 1.0 pg/mL	1.5 pg/mL	71.43 Medium	90.91 High
Carpagnano et al., 2013 [36]	Italy	10 (4;6)	52 ± 5.9	EcoScreen (Jaeger, Wurzburg, Germany)	−20 °C	−70 °C	EIA (Cayman Chemical, Ann Arbor, MI, USA)	15.2 ± 1.9 pg/mL	1.5 pg/mL	71.43 Medium	90.91 High
Carpagnano et al., 2013 [36]	Italy	10 (5;5)	67 ± 4.6	EcoScreen (Jaeger, Wurzburg, Germany)	−20 °C	−70 °C	EIA (Cayman Chemical, Ann Arbor, MI, USA)	16.3 ± 1.4 pg/mL	1.5 pg/mL	71.43 Medium	90.91 High
De lima et al., 2013 [37]	Brazil	73 (73;0)	42 ± 7	EcoScreen (Jaeger, Wurzburg, Germany)	NA	−80 °C	High sensitivity enzyme-immunoassays (Quantikine HS, R&D Systems Inc. Minneapolis, MN, USA)	8.9 ± 1.8 pg/mL	3.50 pg/mL	85.71 High	81.82 High
De lima et al., 2013 [37]	Brazil	14 (14;0)	30 ± 5	EcoScreen (Jaeger, Wurzburg, Germany)	NA	−80 °C	High sensitivity enzyme-immunoassays (Quantikine HS, R&D Systems Inc. Minneapolis, MN, USA)	8.4 ± 0.9 pg/mL	3.50 pg/mL	85.71 High	81.82 High
Guan et al., 2018 [44]	China	15 (7;8)	20 ± 1	ECOScreen (Jager, Germany)	NA	−80 °C	BD Cytometric Bead Array, BD-Biosciences, San Jose, CA, USA	3.58 (1.95) pg/mL	2.4 pg/mL	84.62 High	54.55 Medium
Guan et al., 2018 [44]	China	15 (7;8)	20 ± 1	ECOScreen (Jager, Germany)	NA	−80 °C	BD Cytometric Bead Array, BD-Biosciences, San Jose, CA, USA	3.15 (1.95) pg/mL	2.4 pg/mL	84.62 High	54.55 Medium
Matsunaga et al., 2006 [47]	Japan	10 (3;7)	34.4 ± 6.6	EcoScreen, (Jaeger, Germany)	−20 °C	−70 °C	Human Inflammation Antibody III (ray Biontec Inc, Norcross, GA, USA)	5.4 ± 1.8 pg/mL	1 pg/mL	57.14 Medium	72.73 Medium
**IL-10**											
De lima et al., 2013 [37]	Brazil	14 (14;0)	30 ± 5	EcoScreen (Jaeger, Wurzburg, Germany)	NA	−80 °C	High sensitivity enzyme-immunoassays (Quantikine HS, R&D Systems Inc. Minneapolis, MN, USA)	1.0 (1.4) pg/mL	0.50 pg/mL	85.71 High	81.82 High
De lima et al., 2013 [37]	Brazil	73 (73;0)	42 ± 7	EcoScreen (Jaeger, Wurzburg, Germany)	NA	−80 °C	High sensitivity enzyme-immunoassays (Quantikine HS, R&D Systems Inc. Minneapolis, MN, USA)	1.2 (1.6) pg/mL	0.5 pg/mL	85.71 High	81.82 High
Edmè et al., 2008 * [39]	France	19 (-;-)	38.3 ± 13.6	EcoScreen (Jaeger, Wurzburg, Germany)	NA	−80 °C	Cytometric Bead Arrays (CBA) Becton Dickinson, San Jose, CA, USA	24.3 (13-492) † pg/mL	5 pg/mL	66.67 Medium	66.67 Medium
**TNF-α**											
Carpagnano et al., 2005_b [31]	Italy	7 (5;2)	42 ± 5	EcoScreen (Jaeger, Wurzburg, Germany)	−20 °C	−70 °C	EIA (Cayman Chemical, Ann Arbor, MI, USA)	4.2 ± 0.6 pg/mL	1.5 pg/mL	77.78 High	90.91 High
De lima et al., 2013 [37]	Brazil	14 (14;0)	30 ± 5	EcoScreen (Jaeger, Wurzburg, Germany)	NA	−80 °C	High sensitivity enzyme-immunoassays (Quantikine HS, R&D Systems Inc. Minneapolis, MN, USA)	0.4 (0.2) pg/mL	0.20 pg/mL	85.71 High	81.82 High
De lima et al., 2013 [37]	Brazil	73 (73;0)	42 ± 7	EcoScreen (Jaeger, Wurzburg, Germany)	NA	−80 °C	High sensitivity enzyme-immunoassays (Quantikine HS, R&D Systems Inc. Minneapolis, MN, USA)	0.5 (0.4) pg/mL	0.106 pg/mL	85.71 High	81.82 High
Edmè et al., 2008 * [39]	France	19 (-;-)	38.3 ± 13.6	EcoScreen (Jaeger, Wurzburg, Germany)	NA	−80 °C	Cytometric Bead Arrays (CBA) Becton Dickinson, San Jose, CA, USA	44.6 (32-91) † pg/mL	5 pg/mL	66.67 Medium	66.67 Medium
Garey et al., 2004 [41]	USA	9 (5;4)	22.0 ± 1.9	Breath condensate was collected using a novel method where the subject inspires repeatedly to TLC and exhales into 1.5 m Teflon perfluoroalkoxy (PFA) tubing with 0.5 cm internal diameter	Immersed in ice	−70 °C	ELISA (R&D System Minneapolis, MN)	3.9 ± 8.5 pg/mL	2 pg/mL	71.43 Medium	54.55 Medium
Guan et al., 2018 [44]	China	15 (7;8)	20 ± 1	ECOScreen (Jager, Germany)	NA	−80 °C	BD Cytometric Bead Array, BD-Biosciences, San Jose, CA, USA	4.36 (1.79) pg/mL	2.4 pg/mL	84.62 High	54.55 Medium
Guan et al., 2018 [44]	China	15 (7;8)	20 ± 1	ECOScreen (Jager, Germany)	NA	−80 °C	BD Cytometric Bead Array, BD-Biosciences, San Jose, CA, USA	4.14 (2.56) pg/mL	2.4 pg/mL	84.62 High	54.55 Medium
Ko et al., 2009 [45]	China	14 (9;5)	75.2 ± 4.1	EcoScreen (VIASYS Healthcare, Conshohochen, PA, USA)	NA	−70 °C	BioSource International, Camarillo, CA, USA	4.84 (3.86-5.81) pg/mL	0.09 pg/mL	71.43 Medium	81.82 High

The various biomarkers analysed are highlighted in bold. (*) In the study of Edmé et al., the concentrations declared were not divided by the concentration factor.

## Data Availability

Not applicable.

## Supplementary Materials

The following supporting information can be downloaded at: https://www.mdpi.com/article/10.3390/ijms23179820/s1.

## Appendix A

**Table A1:** Search strings.

*PubMed*	
1	“Tumor Necrosis Factor-alpha” [Mesh]
2	“tumor necrosis factor-alpha” [tiab] OR “tumor necrosis factor-a” [tiab] OR “TNF-alpha” [tiab] OR TNFalpha [tiab] OR TNF-a [tiab] OR TNFa [tiab] OR “tumor necrosis factor (TNF)-alpha” [tiab]
3	“C-Reactive Protein” [Mesh]
4	“C-Reactive Protein” [tiab] OR CRP [tiab]
5	“Cytokines” [MESH:noexp]
6	“Interleukins” [MESH:noexp]
7	cytokines [tiab] OR interleukins [tiab]
8	“Interleukin-1” [Mesh]
9	“interleukin-1beta” [tiab] OR “interleukin-1 beta” [tiab] OR “interleukin-1 b” [tiab] OR “interleukin-1b” [tiab] OR “IL-1beta” [tiab] OR “IL-1 beta” [tiab] OR “IL1beta” [tiab] OR “IL1 beta” [tiab] OR “IL-1b” [tiab] OR “IL-1 b” [tiab] OR “IL1b” [tiab] OR “IL1 b” [tiab] OR “interleukin (IL)-1beta” [tiab] OR “interleukin (IL)-1 beta” [tiab]
10	“Interleukin-4” [Mesh]
11	“interleukin-4” [tiab] OR “IL-4” [tiab] OR IL4 [tiab] OR “interleukin (IL)-4” [tiab]
12	“Interleukin-6” [Mesh]
13	“interleukin-6” [tiab] OR “IL-6” [tiab] OR IL6 [tiab] OR “interleukin (IL)-6” [tiab]
14	“Interleukin-8” [Mesh]
15	“interleukin-8” [tiab] OR “IL-8” [tiab] OR IL8 [tiab] OR “interleukin (IL)-8” [tiab]
16	“Interleukin-10” [Mesh]
17	“interleukin-10” [tiab] OR “IL-10” [tiab] OR IL10 [tiab] OR “interleukin (IL)-10” [tiab]
18	#1 OR #2 OR #3 OR #4 OR #5 OR #6 OR #7 OR #8 OR #9 OR #10 OR #11 OR #12 OR #13 OR #14 OR #15 OR #16 OR #17
19	“exhaled breath condensate *” [tiab] OR EBC [tiab] OR EBCs [tiab] OR “exhaled breath” [tiab] OR “breath condensate *” [tiab]
20	“Breath Tests” [Mesh]
21	“Exhalation” [Mesh]
22	#19 OR #20 OR #21
23	#18 AND #22
24	“Animals” [Mesh]
25	“Humans” [Mesh]
26	#24 NOT #25
27	#23 NOT #26
28	“Adolescent” [Mesh]
29	“Child” [Mesh]
30	“Infant” [Mesh]
31	#28 OR #29 OR #30
32	“Adult” [Mesh]
33	#31 NOT #32
34	#27 NOT #33
***Embase***	
1	‘tumor necrosis factor’/exp
2	‘tumor necrosis factor-alpha’:ti,ab,kw OR ‘tumor necrosis factor-a’:ti,ab,kw OR ‘TNF-alpha’:ti,ab,kw OR TNFalpha:ti,ab,kw OR TNF-a:ti,ab,kw OR TNFa:ti,ab,kw OR ‘tumor necrosis factor (TNF)-alpha’:ti,ab,kw
3	‘C reactive protein’/exp
4	‘C-Reactive Protein’:ti,ab,kw OR CRP:ti,ab,kw
5	‘cytokine’/de
6	‘interleukin derivative’/de
7	cytokines:ti,ab,kw OR interleukins:ti,ab,kw
8	‘interleukin 1’/exp
9	‘interleukin-1beta’:ti,ab,kw OR ‘interleukin-1 beta’:ti,ab,kw OR ‘interleukin-1 b’:ti,ab,kw OR ‘interleukin-1b’:ti,ab,kw OR ‘IL-1beta’:ti,ab,kw OR ‘IL-1 beta’:ti,ab,kw OR ‘IL1beta’:ti,ab,kw OR ‘IL1 beta’:ti,ab,kw OR ‘IL-1b’:ti,ab,kw OR ‘IL-1 b’:ti,ab,kw OR ‘IL1b’:ti,ab,kw OR ‘IL1 b’:ti,ab,kw OR ‘interleukin (IL)-1beta’:ti,ab,kw OR ‘interleukin (IL)-1 beta’:ti,ab,kw
10	‘interleukin 4’/exp
11	‘interleukin-4′:ti,ab,kw OR ‘IL-4′:ti,ab,kw OR ‘IL4′:ti,ab,kw OR ‘interleukin (IL)-4′:ti,ab,kw
12	‘interleukin 6’/exp
13	‘interleukin-6′:ti,ab,kw OR ‘IL-6′:ti,ab,kw OR ‘IL6′:ti,ab,kw OR ‘interleukin (IL)-6′:ti,ab,kw
14	‘interleukin 8’/exp
15	‘interleukin-8′:ti,ab,kw OR ‘IL-8′:ti,ab,kw OR ‘IL8′:ti,ab,kw OR ‘interleukin (IL)-8′:ti,ab,kw
16	‘interleukin 10’/exp
17	‘interleukin-10′:ti,ab,kw OR ‘IL-10′:ti,ab,kw OR ‘IL10′:ti,ab,kw OR ‘interleukin (IL)-10′:ti,ab,kw
18	#1 OR #2 OR #3 OR #4 OR #5 OR #6 OR #7 OR #8 OR #9 OR #10 OR #11 OR #12 OR #13 OR #14 OR #15 OR #16 OR #17
19	‘exhaled breath condensate’/exp
20	‘exhaled breath condensate *’:ti,ab,kw OR EBC:ti,ab,kw OR EBCs:ti,ab,kw OR ‘exhaled breath’:ti,ab,kw OR ‘breath condensate *’:ti,ab,kw
21	‘breath analysis’/exp
22	‘exhalation’/exp
23	#19 OR #20 OR #21 OR #22
24	#18 AND #23
25	‘animal’/de
26	‘animal experiment’/exp
27	‘nonhuman’/de
28	#25 OR #26 OR #27
29	‘human’/de
30	#28 NOT #29
31	#23 NOT #30
32	‘adolescent’/exp
33	‘child’/exp
34	#32 OR #33
35	‘adult’/exp
36	#34 NOT #35
37	#31 NOT #36
***Cochrane CENTRAL***	
#1	MeSH descriptor: [Tumor Necrosis Factor-alpha] explode all trees
#2	(“tumor necrosis factor-alpha” OR “tumor necrosis factor-a” OR “TNF-alpha” OR TNFalpha OR TNF-a OR TNFa):ti,ab,kw
#3	MeSH descriptor: [C-Reactive Protein] explode all trees
#4	(“C-Reactive Protein” OR CRP):ti,ab,kw
#5	MeSH descriptor: [Cytokines] this term only
#6	MeSH descriptor: [Interleukins] this term only
#7	(cytokines OR interleukins):ti,ab,kw
#8	MeSH descriptor: [Interleukin-1] explode all trees
#9	(“interleukin-1beta” OR “interleukin-1 beta” OR “interleukin-1 b” OR “interleukin-1b” OR “IL-1beta” OR “IL-1 beta” OR “IL1beta” OR “IL1 beta” OR “IL-1b” OR “IL-1 b” OR “IL1b” OR “IL1 b”):ti,ab,kw
#10	MeSH descriptor: [Interleukin-4] explode all trees
#11	(“interleukin-4” OR “IL-4” OR IL4):ti,ab,kw
#12	MeSH descriptor: [Interleukin-6] explode all trees
#13	(“interleukin-6” OR “IL-6” OR IL6):ti,ab,kw
#14	MeSH descriptor: [Interleukin-8] explode all trees
#15	(“interleukin-8” OR “IL-8” OR IL8):ti,ab,kw
#16	MeSH descriptor: [Interleukin-10] explode all trees
#17	(“interleukin-10” OR “IL-10” OR IL10):ti,ab,kw
#18	#1 OR #2 OR #3 OR #4 OR #5 OR #6 OR #7 OR #8 OR #9 OR #10 OR #11 OR #12 OR #13 OR #14 OR #15 OR #16 OR #17
#19	(“exhaled breath condensate *” OR EBC OR EBCs OR “exhaled breath” OR “breath condensate *”):ti,ab,kw
#20	MeSH descriptor: [Breath Tests] explode all trees
#21	MeSH descriptor: [Exhalation] explode all trees
#22	#19 OR #20 OR #21
#23	#18 AND #22.

(*) The asterisk was employed to retrieve any variations of the indicated terms.

## Appendix B

**Table A2:** Authors quality assessment additional questions.

1	Was the type of EBC sampler used specified?
2	Was the EBC collection temperature between −10 °C and −20 °C?
3	Was the duration of condensation specified?
4	Were the ventilation patterns such as the breathing frequencies specified?
5	Did subjects wear a noseclip?
6	Was any precaution taken to avoid saliva contamination of EBC samples?
7	Were samples stored at ≤−70 °C?
8	Was the storage duration specified?
9	Have the assay characteristics used for analysis been specified?
10	Have lower limits of detection (LODs) been given?
11	Were intra- and inter-variability of the assay specified?
12	Were appropriate data on recovery in case of sample concentration specified?
