# Supplementary material for: Inflammatory Biomarkers in Exhaled Breath Condensate: A Systematic Review
Source: Int J Mol Sci. 2022 Aug 29;23(17):9820. doi: 10.3390/ijms23179820 (PMC9456215; doi:10.3390/ijms23179820)
Supplement: Supplementary file 1 [file ijms-23-09820-s001.zip › ijms-1859831-supplementary.pdf]

Table S1. Data extracted from articles reporting data below the declared assay LOD or not declaring the assay LOD. *Data are expressed as: Geometric mean =†; Mean ± SD; Median (IQR); Median ("25°th – 75°th"); Median [min-max].*

AUTHORS, YEAR	Country	n° subjects (M;F)	Age	Collection Device	Collection Temperature	Storage Temperature	Analytical Method	Data	LOD	SCORE Quality Assessment JBI	SCORE Authors' Quality Assessment
**CRP**											
**NO LOD**											
YAN ET AL., 2019 [57]	China	57 (7;50)	60±9.5	NA	NA	NA	immune non equilibrium method	14	NA	66.67 Medium	0 Low
**IL-1β**											
**< LOD**											
DE LIMA ET AL., 2013 [37]	Brazil	73 (73;0)	42±7	RT-Tube (Respiratory Research, USA) or ECOScreen	-20°C	-80°C	High sensitivity enzyme-immunoassays (Quantikine HS, R&D Systems Inc. Minneapolis, USA)	0 (7)	0.057 pg/mL	85.71 High	81.82 High
DE LIMA ET AL., 2013 [37]	Brazil	14 (14;0)	30±5	RT-Tube (Respiratory Research, USA) or ECOScreen	-20°C	-80°C	High sensitivity enzyme-immunoassays (Quantikine HS, R&D Systems Inc. Minneapolis, USA)	0 (0)	0.057 pg/mL	85.71 High	81.82 High
GAREY ET AL., 2004 [41]	USA	9 (5;4)	22±1.9	Breath condensate was collected using a novel method	Immersed in ice	-70°C	ELISA (R&D System	1.5±1.0	2 pg/mL	71.43 Medium	54.55 Medium

				where the subject inspires repeatedly to TLC and exhales into 1.5 m Teflon perfluoroalkoxy (PFA) tubing with 0.5 cm internal diameter			Minneapolis, MN)				
Matsunaga et al., 2006 [47]	Japan	10 (3;7)	34±6.6	EcoScreen (Jaeger, Germany)	-20°C	-70°C	Human Inflammation Antibody III (ray Biontec Inc, Norcross, Ga)	4.6±0.9	100 pg/mL	71.43 Medium	63.64 Medium
Sack et al., 2006 [53]	Germany	21 (11;10)	58±13	Ecoscreen (ViaSys, Hoechberg, Germany) FILT Lung and Chest Diagnostics Ltd., Berlin, Germany	NA	NA	Multifluorescent bead array CBA , BD-Biosciences, San Jose, CA, USA	0.06 (1.45)	0.15 pg/mL	57.14 Medium	45.45 Low
Tufvesson et al., 2006 [55]	Sweden	12 (-;-)	35 (19-53)	EcoScreen (Jaeger, Wurzburg, Germany)	NA	-80°C	ELISA (R&D Systems, Abingdon, UK)	n.d. ±n.d.	0.1 pg/mL	66.67 Medium	66.67 Medium
					**NO LOD**						
Ausin et al., 2017 [25]	Spain	8 (0;8)	62±8.2	EcoScreen/ECoVent, Jaeger, Germany	NA	NA	ELISA kits (RayBiotech, Norcross, Georgia, USA)	26±18	/	85.71 High	27.27 Low
Gessner et al., 2005 [42]	Germany	24 (13;11)	54.7 ±10.5	Ecoscreen device (Viasys, Hechberg, Germany)	-20°C	NA	Cytometric bead array [CBA] Becton Dickinson, San Jose, CA, USA	2.60 (12.07)	/	57.14 Medium	66.67 Medium

GESSNER ET AL., 2007 [43]	Germany	17 (11;6)	60.5 ±14.7	Ecoscreen device (Viasys, Hechberg, Germany)	-20°C	NA	Cytometric bead array [CBA] Becton Dickinson, San Jose, CA, USA	5.77 (8.33)	/	57.14 Medium	66.67 Medium
MAYSA ALVES RODRIGUES BRANDAO-RANGEL ET AL., 2021 [26]	Brazil	77 (23;54)	68 (63-73)	RT-Tube (Respiratory Research, USA)	NA	-86°C	ELISA (SpectraMax i3, Molecular Devices, USA)	3.22±0.78	/	71.43 Medium	54.55 Medium
ROLLA ET AL., 2016 [52]	Italy	20 (8;12)	59 [47-65]	R-Tube (Respiratory Research, Inc., Charlottesville, VA, USA)	-20°C	-80°C	Multiplex immunoassay (Bio-Rad Laboratories Inc., Hercules, CA, USA)	0.23 (0.02-0.91)	/	57.14 Medium	72.73 Medium
**IL-4**											
**NO LOD**											
AUSIN ET AL., 2017 [25]	Spain	8 (0;8)	62±8.2	EcoScreen/ECoVent, Jaeger, Germany	NA	NA	ELISA kits (RayBiotech, Norcross, Georgia, USA)	189±10	/	85.71 High	27.27 Low
FONT-RIBERA ET AL., 2010 [40]	Spain	48 (17;31)	30±6.1	EcoScreen condenser (jager GmbH, Wurzburg, Germany)	NA	-80°C	BD Cytometric Bead Array (CBA; BD Biosciences, Erembodegem, Belgium) and BD FACSCalibur Flow Cytometer (Becton Dickinson, San Jose, CA, USA)	0.70 (0.3-2.0)	/	87.50 High	45.45 Low

LIU ET AL., 2014 [46]	Taiwan	10 (5;5)	44±8.5	Custom-made condensing device	+4°C	-70°C	ELISA (eBioscience, San Diego, CA)	0.02±0.02	/	71.43 Medium	36.36 Low
					**IL-6**						
					**<LOD**						
NIELEPKOWICZ-GOŹDZIŃSKA ET AL., 2013 [49]	Poland	31 (4;27)	31.2 ±4.5	EcoScreen (Jaeger)	NA	-80°C	EIA (Quantikine, R&D Systems, United States)	n.d.	0.3 pg/mL	57.14 Medium	63.64 Medium
SACK ET AL., 2006 [53]	Germany	21 (11;10)	58±13	Ecoscreen (ViaSys, Hoechberg, Germany) FILT Lung and Chest Diagnostics Ltd., Berlin, Germany	NA	NA	Multifluorescent bead array CBA , BD-Biosciences, San Jose, CA, USA	0.021 (0.37)	0.15 pg/mL	57.14 Medium	45.45 Low
					**NO LOD**						
AUSIN ET AL., 2017 [25]	Spain	8 (0;8)	62±8.2	EcoScreen/ECoVent, Jaeger, Germany	NA	NA	ELISA kits (RayBiotech, Norcross, Georgia, USA)	23±12	/	85.71 High	27.27 Low
GESSNER ET AL., 2005 [42]	Germany	24 (13;11)	54.7 ±10.5	Ecoscreen device (Viasys, Hechberg, Germany)	-20°C	NA	Cytometric bead array [CBA] Becton Dickinson, San Jose, CA, USA	2.60 (12.07)	/	57.14 Medium	66.67 Medium
GESSNER ET AL., 2007 [43]	Germany	17 (11;6)	60.5 ±14.7	Ecoscreen device (Viasys, Hechberg, Germany)	-20°C	NA	Cytometric bead array [CBA] Becton Dickinson, San Jose, CA, USA	0.16 (4.54)	/	57.14 Medium	66.67 Medium
RADULOVIC ET AL., 2015 [51]	USA	10 (10;0)	48±10	EcoScreen EBC collector (Jaeger,	-10°C	-80°C	EIA kits (Cayman Chemical, Ann	4.9±3.8	/	75.00 High	45.45 Low

				Wurzburg, Germany)			Arbor, Michigan)				
ROLLA ET AL., 2016 [52]	Italy	20 (8;12)	59 [47-65]	R-Tube (Respiratory Research, Inc., Charlottesville, VA, USA)	-20°C	-80°C	Multiplex immunoassay (Bio-Rad Laboratories Inc., Hercules, CA, USA)	0.19 (0.13-0.34)	/	57.14 Medium	72.73 Medium
VERGARA ET AL., 2015 [56]	Chile	8 (3;5)	22±1.1	EcoScreen (Jaeger, Wurzburg, Germany)	-20°C	-70°C	human IL-6 Elisa kit (Pierce, USA)	5.71;3.59-7.82 CI 95%	/	83.33 High	90.91 High
**IL-8**											
**<LOD**											
KO ET AL., 2009 [45]	China	14 (9;5)	75.2 ±4.1	EcoScreen (VIASYS Healthcare, Conshohochen, PA, USA)	NA	-70°C	BioSource International, Camarillo, CA, USA	n.a. ± n.a.	0.02 pg/mL	71.43 Medium	81.82 High
NIELEPKOWICZ-GOŹDZIŃSKA ET AL., 2014 [50]	Poland	31 (4;27)	31.2 ±4.5	EcoScreen (Jaeger)	NA	-80°C	EIA (Quantikine, R&D Systems, United States)	1.7±1.7	3.4 pg/mL	71.43 Medium	63.64 Medium
SACK ET AL., 2006 [53]	Germany	21 (11;10)	58±13	Ecoscreen (ViaSys, Hoechberg, Germany) FILT Lung and Chest Diagnostics Ltd., Berlin, Germany	NA	NA	Multifluorescent bead array CBA , BD-Biosciences, San Jose, CA, USA	0.05 (0.55)	0.15 pg/mL	57.14 Medium	45.45 Low
TUFVESSON ET AL., 2006 [55]	Sweden	12 (-;-)	35 (19-53)	EcoScreen (Jaeger, Wurzburg, Germany)	NA	-80°C	ELISA (R&D Systems, Abingdon, UK)	n.d. ±n.d.	3.5 pg/mL	66.67 Medium	66.67 Medium
**NO LOD**											
AUSIN ET AL., 2017 [25]	Spain	8 (0;8)	62±8.2	EcoScreen/	NA	NA	ELISA kits (RayBiotech,	79±5	/	85.71 High	27.27 Low

				ECoVent, Jaeger, Germany			Norcross, Georgia, USA)				
FONT-RIBERA ET AL., 2010 [40]	Spain	48 (17;31)	30±6.1	EcoScreen condenser (jager GmbH, Wurzburg, Germany)	NA	-80°C	BD Cytometric Bead Array (CBA; BD Biosciences, Erembodegem, Belgium) and BD FACSCalibur Flow Cytometer (Becton Dickinson, San Jose, CA, USA)	1.24 (0.7-2.2)	/	87.50 High	45.45 Low
GESSNER ET AL., 2005 [42]	Germany	24 (13;11)	54.7±10.5	Ecoscreen device (Viasys, Hechberg, Germany)	-20°C	NA	Cytometric bead array [CBA] Becton Dickinson, San Jose, CA, USA	1.47 (7.36)	/	57.14 Medium	66.67 Medium
GESSNER ET AL., 2007 [43]	Germany	17 (11;6)	60.5 ±14.7	Ecoscreen device (Viasys, Hechberg, Germany)	-20°C	NA	Cytometric bead array [CBA] Becton Dickinson, San Jose, CA, USA	3.66 (5.90)	/	57.14 Medium	66.67 Medium
LIU ET AL., 2014 [46]	Taiwan	10 (5;5)	44±8.5	Custom-made condensing device	+4°C	-70°C	ELISA (eBioscience, San Diego, CA)	4.9±0.05	/	71.43 Medium	36.36 Low
MAYSA ALVES RODRIGUES BRANDAO-RANGEL ET AL., 2021 [26]	Brazil	77 (23;54)	68 (63-73)	RT-Tube (Respiratory Research, USA)	NA	-86°C	ELISA (SpectraMax i3, Molecular Devices, USA)	41.22±2.291	/	71.43 Medium	54.55 Medium
MAZUR ET AL., 2009 [48]	Finland	14 (11;3)	57±4.2	Ecoscreen Version 1.1; jager,	-20°C	-80°C	Enzyme - Amplified	n.a. ±n.a.	/	100.0 High	45.45 Low

				Wurzburg, Germany			Sensitivity Immunoassay (EASIA) (BioSource SA, Nivelles, Belgium)				
					**IL-10**						
					**<LOD**						
MATSUNAGA ET AL., 2006 [47]	Japan	10 (3;7)	34±6.6	EcoScreen, Jaeger, German	-20°C	-70°C	Human Inflammation Antibody III (ray Biontec Inc, Norcross, Ga)	5.4±1.8	10 pg/mL	71.43 Medium	63.64 Medium
SACK ET AL., 2006 [53]	Germany	21 (11;10)	58±13	Ecoscreen (ViaSys, Hoechberg, Germany) FILT Lung and Chest Diagnostics Ltd., Berlin, Germany	NA	NA	Multifluorescent bead array CBA , BD-Biosciences, San Jose, CA, USA	0.06 (1.24)	0.15 pg/mL	57.14 Medium	45.45 Low
					**NO LOD**						
AQUINO-SANTOS ET AL., 2020 [24]	Brazil	25 (-;-)	34±9.6	RT Tube (Respiratory Research, TX, USA)	NA	-86°C	DuoSet ELISA kit (R&D Systems)	33.4±4.17	/	75.00 High	33.33 Low
AQUINO-SANTOS ET AL., 2020 [24]	Brazil	25 (-;-)	38.73±6.92	RT Tube (Respiratory Research, TX, USA)	NA	-86°C	DuoSet ELISA kit (R&D Systems)	27.08±2.36	/	75.00 High	33.33 Low
AUSIN ET AL., 2017 [25]	Spain	8 (0;8)	62±8.2	EcoScreen/ ECoVent, Jaeger, Germany	NA	NA	ELISA kits (RayBiotech, Norcross, Georgia, USA)	101±15	/	85.71 High	27.27 Low
FONT-RIBERA ET AL., 2010 [40]	Spain	48 (17;31)	30±6.1	EcoScreen condenser (jager	NA	-80°C	BD Cytometric Bead Array (CBA; BD	0.89 (0.2-1.5)	/	87.50 High	45.45 Medium

				GmbH, Wurzburg, Germany)			Biosciences, Erembodegem, Belgium) and BD FACSCalibur Flow Cytometer (Becton Dickinson, San Jose, CA, USA)				
Gessner et al., 2005 [42]	Germany	24 (13;11)	54.7 ±10.5	Ecoscreen device (Viasys, Hechberg, Germany)	-20°C	NA	Cytometric bead array [CBA] Becton Dickinson, San Jose, CA, USA	1.70 (9.27)	/	57.14 Medium	66.67 Medium
Gessner et al., 2007 [43]	Germany	17 (11;6)	60.5 ±14.7	Ecoscreen device (Viasys, Hechberg, Germany)	-20°C	NA	Cytometric bead array [CBA] Becton Dickinson, San Jose, CA, USA	7.18 (29.12)	/	57.14 Medium	66.67 Medium
Maysa Alves Rodrigues Brandao-Rangel et al., 2021 [26]	Brazil	77 (23;54)	68 (63-73)	RT-Tube (Respiratory Research, USA)	NA	-86°C	ELISA (SpectraMax i3, Molecular Devices, USA)	24.19±1.36	/	71.43 Medium	54.55 Medium
Nielepkowicz-Goździńska et al., 2013 [49]	Poland	31 (4;27)	31.2 ±4.5	EcoScreen (Jaeger)	NA	-80°C	EIA (Quantikine, R&D Systems, United States)	0 ± 1.68	/	57.14 Medium	63.64 Medium
Rolla et al., 2016 [52]	Italy	20 (8;12)	59 [47-65]	R-Tube (Respiratory Research, Inc., Charlottesville, VA, USA)	-20°C	-80°C	Multiplex immunoassay (Bio-Rad Laboratories Inc., Hercules, CA, USA)	1.00 (0.25)	/	57.14 Medium	72.73 Medium

**TNF-α**											
**<LOD**											
Carpagnano et al., 2013 [36]	Italy	10 (5;5)	26±4.9	EcoScreen (Jaeger, Wurzburg, Germany)	-20°C	-70°C	EIA (Cayman Chemical, Ann Arbor, MI, USA)	19.8±3.0	1 ng/mL	71.43 Medium	90.91 High
Carpagnano et al., 2013 [36]	Italy	10 (4;6)	52±5.9	EcoScreen (Jaeger, Wurzburg, Germany)	-20°C	-70°C	EIA (Cayman Chemical, Ann Arbor, MI, USA)	23.9±1.6	1 ng/mL	71.43 Medium	90.91 High
Carpagnano et al., 2013 [36]	Italy	10 (5;5)	67±4.6	EcoScreen (Jaeger, Wurzburg, Germany)	-20°C	-70°C	EIA (Cayman Chemical, Ann Arbor, MI, USA)	24.1±1.7	1 ng/mL	71.43 Medium	90.91 High
Matsunaga et al., 2006 [47]	Japan	10 (3;7)	34±6.6	EcoScreen, Jaeger, Germany	-20°C	-70°C	Human Inflammation Antibody III (ray Biontec Inc, Norcross, Ga)	7.0±1.0	50 pg/mL	71.43 Medium	63.64 Medium
Sack et al., 2006 [53]	Germany	21 (11;10)	58±13	Ecoscreen (ViaSys, Hoechberg, Germany) FILT Lung and Chest Diagnostics Ltd., Berlin, Germany	NA	NA	Multifluorescent bead array CBA , BD-Biosciences, San Jose, CA, USA	0.053 (0.29)	0.15 pg/mL	57.14 Medium	45.45 Low
**NO LOD**											
Ausin et al., 2017 [25]	Spain	8 (0;8)	62±8.2	EcoScreen/ ECoVent, Jaeger, GermanY	NA	NA	ELISA kits (RayBiotech, Norcross, Georgia, USA)	156±8	/	85.71 High	27.27 Low
Diez-Pina et al., 2009 [38]	Spain	9 (5;4)	35.1 ±6.56	EcoScreen (Jaeger)	NA	-70°C	ELISA (DRG Diagnostics	4.34 (0.62)	/	75.00 High	45.45 Low

							GMBH, Germany)				
Diez-Pina et al., 2009 [38]	Spain	5 (5;0)	NA	EcoScreen (Jaeger)	NA	-70°C	ELISA (DRG Diagnostics GMBH, Germany)	4.22 (0.36)	/	75.00 High	45.45 Low
Diez-Pina et al., 2009 [38]	Spain	4 (0;4)	NA	EcoScreen (Jaeger)	NA	-70°C	ELISA (DRG Diagnostics GMBH, Germany)	4.11 (0.0.41)	/	75.00 High	45.45 Low
Font-Ribera et al., 2010 [40]	Spain	48 (17;31)	30±6.1	EcoScreen condenser (jager GmbH, Wurzburg, Germany)	NA	-80°C	BD Cytometric Bead Array (CBA; BD Biosciences, Erembodegem, Belgium) and BD FACSCalibur Flow Cytometer (Becton Dickinson, San Jose, CA, USA)	0.89 (0.4-1.7)	/	87.5 High	45.45 Low
Gessner et al., 2005 [42]	Germany	24 (13;11)	54.7 ±10.5	Ecoscreen device (Viasys, Hechberg, Germany)	-20°C	NA	Cytometric bead array [CBA] Becton Dickinson, San Jose, CA, USA	1.57 (3.97)	/	57.14 Medium	66.67 Medium
Gessner et al., 2007 [43]	Germany	17 (11;6)	60.5 ±14.7	Ecoscreen device (Viasys, Hechberg, Germany)	-20°C	NA	Cytometric bead array [CBA] Becton Dickinson, San Jose, CA, USA	3.54 (3.23)	/	57.14 Medium	66.67 Medium

LIU ET AL., 2014 [46]	Taiwan	10 (5;5)	44±8.5	Custom-made condensing device	+4°C	-70°C	ELISA (eBioscience, San Diego, CA)	0.21±0.04	/	71.43 Medium	36.36 Low
MAYSA ALVES RODRIGUES BRANDAO-RANGEL ET AL., 2021 [26]	Brazil	77 (23;54)	68 (63-73)	RT-Tube (Respiratory Research, USA)	NA	-86°C	ELISA (SpectraMax i3, Molecular Devices, USA)	4.57±1.5	/	71.43 Medium	54.55 Medium
ROLLA ET AL., 2016 [52]	Italy	20 (8;12)	59 [47-65]	R-Tube (Respiratory Research, Inc., Charlottesville, VA, USA)	-20°C	-80°C	Multiplex immunoassay (Bio-Rad Laboratories Inc., Hercules, CA, USA)	0.75 (0.4)	/	57.14 Medium	72.73 Medium
RADULOVIC ET AL., 2015 [51]	USA	10 (10;0)	48±10	EcoScreen EBC collector (Jaeger, Wurzburg, Germany)	-10°C	-80°C	EIA kits (Cayman Chemical, Ann Arbor, Michigan)	5.6±5.9	/	75.00 High	45.45 Low
